# The Interaction of CuS and *Halothiobacillus* HT1 Biofilm in Microscale Using Synchrotron Radiation-Based Techniques

**DOI:** 10.3390/ijms140611113

**Published:** 2013-05-24

**Authors:** Huirong Lin, Guangcun Chen, Shenhai Zhu, Yingxu Chen, Dongliang Chen, Wei Xu, Xiaohan Yu, Jiyan Shi

**Affiliations:** 1 Department of Environmental Engineering, Zhejiang University, Hangzhou 310058, China; E-Mails: hrlin@iue.ac.cn (H.L.); gcchen2011@sinano.ac.cn (G.C.); zhushenhai@yahoo.com.cn (S.Z.); yingxu_chen@hotmail.com (Y.C.); 2 Institute of Urban Environment, Chinese Academy of Science, Xiamen 361021, China; 3 Institute of High Energy Physics, Chinese Academy of Science, Beijing Synchrotron Radiation Facility, Beijing 100049, China; E-Mails: chendl@ihep.ac.cn (D.C.); xuw@mail.ihep.ac.cn (W.X.); 4 Institute of Applied Physics, Chinese Academy of Science, Shanghai Synchrotron Radiation Facility, Shanghai 201800, China; E-Mail: yuxiaohan@sinap.ac.cn

**Keywords:** *Halothiobacillus* HT1 biofilm, distribution, speciation, copper, sulfur

## Abstract

In order to investigate the microbe-mineral interaction in the micro scale, spatial distribution and speciation of Cu and S in *Halothiobacillus* HT1 biofilm formed on a CuS surface was examined using synchrotron-based X-ray techniques. Confocal laser scanning microscope (CLSM) results indicated that *Halothiobacillus* HT1 biofilm formation gave rise to distinct chemical and redox gradients, leading to diverse niches in the biofilm. Live cells were distributed at the air-biofilm and membrane-biofilm interface. CuS was oxidized by *Halothiobacillus* HT1 biofilm, and copper penetrated into the biofilm. Sulfide was oxidized to cysteine (77.3%), sulfite (3.8%) and sulfonate (18.9%). Cu-cysteine-like species were involved in the copper homeostasis. These results significantly improve our understanding of the interfacial properties of the biofilm-mineral interface.

## 1. Introduction

Microbes cycle metals through biogeochemical processes, including accumulation, transformation and biomineralization, thus leading to changes in toxicity and bioavailability [1,2]. Sulfide oxidation by microbes is used for the biological production of metals, such as copper, gold and zinc. Sulfur oxidizing bacteria contribute strongly to the biological oxidation of metal sulfide [3,4]. However, contrary to their significant role in the global sulfur cycle and the biotechnological importance, the microbial fundamentals of sulfur oxidation are incompletely understood, due to the complexity of this reaction.

The microbe-mineral interface serves as a solid phase source of electrons, and biogeochemistry of the microbe-mineral interactions have been paid great attention to during the past few decades [5–7]. Many of the critical processes occur at the biofilm-mineral interface on the molecular scale. Therefore, attention should be focused on microenvironments, where chemical transformations occur. A better understanding of the interfacial properties of the biofilms-mineral interface, especially the interfacial chemical processes at the micron and nanometer levels, is needed [6,8].

Microbes usually operate as consortia of organisms rather than as single cells. Biofilms are physiologically distinct from bacteria growing in a free-swimming planktonic state and present genetic and physiological heterogeneity [9–11]. Growth of biofilm can enhance resistance to metal toxicity. Previous report showed that the ability of biofilms to survive heavy metals stress is better than planktonic microbes [12]. The metabolic activity, microenvironment characteristics and microbial community composition of biofilm are involved in resistance to metal toxicity. Biofilms can sorb metals and retard metal diffusion, leading to protection in the interior of the biofilms [13]. The genetic basis for metal resistance in sulfur oxidizing bacteria has been studied by several investigators [14]. Basic understanding of environmental materials and processes at the molecular scale is essential for risk assessment and management and reduction of environmental pollutants. Therefore, the description of the speciation and distribution of metals in biofilms is critically important for modeling and understanding the detoxification mechanism.

The aim of this study was to investigate the interaction in sulfur oxidizing bacteria biofilm-metal sulfide at the micro scale. A heavy-metals-tolerant *Halothiobacillus* HT1 was chosen [15]. The interaction in the CuS and biofilm of the *Halothiobacillus* interface was studied. The spatial distribution and speciation of copper in *Halothiobacillus* HT1 biofilm formed on CuS was determined using synchrotron-based X-ray fluorescence microscopy (XRF) and micro-X-ray absorption near edge structure (micro-XANES) analysis. Cell viability was detected using live-dead staining. Sulfur speciation was measured using sulfur K-edge XANES.

## 2. Results

### 2.1. Spatial Distribution of Live and Dead *Halothiobacillus* HT1 Cells in the Biofilms

**Figure 1:**
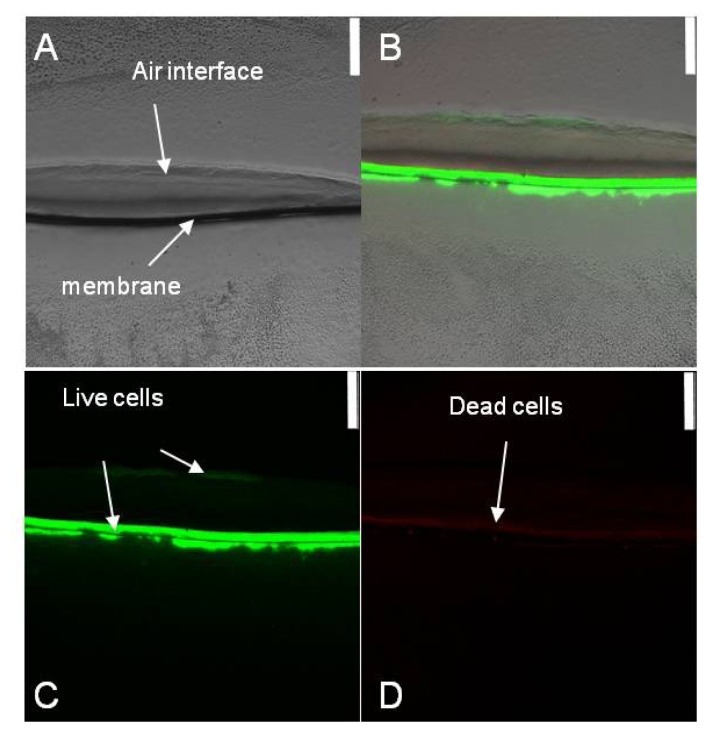
Spatial distribution of live and dead cells in *Halothiobacillus* HT1 biofilm reacted with CuS after dyeing and CLSM. (**A**) Light microscope images of *Halothiobacillus* HT1 biofilm; (**B**) a composite of live and dead cells; (**C**) live cells; (**D**) dead cells. Bars = 250 μm.

*Halothiobacillus* HT1 is a heavy-metals-tolerant sulfur oxidizing bacterium, which belongs to Gammaproteobacteria, *Halothiobacillus* (GenBank accession number GU013549). In order to analyze the interaction of *Halothiobacillus* HT1 biofilm and CuS, the spatial distribution of live and dead cells in *Halothiobacillus* HT1 biofilm sections was studied using Live/Dead staining combined with CLSM imaging. As shown in Figure 1, after 72 h cultivation, the HT1 cells presented different distributions in the biofilm formed on CuS. At the air-biofilm interface and membrane-biofilm interface, the CLSM imaging results showed green, while in the middle, the results showed red. These results indicated that there were more live cells at the air-biofilm interface and membrane-biofilm interface than in the middle.

### 2.2. Spatial Distributions of Cu in Biofilm and Cu Speciation

**Figure 2:**
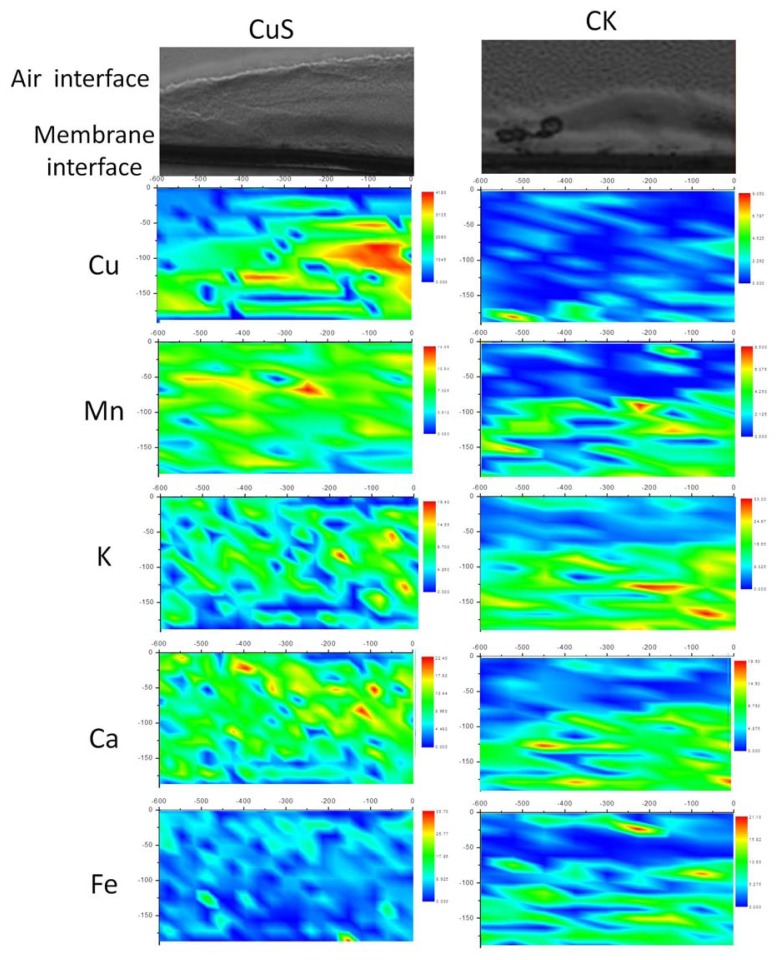
X-ray fluorescence microscopy (XRF) maps of qualitative spatial distributions and concentration gradients of elements in the sections of *Halothiobacillus* HT1 biofilms grown on LB medium. (**Left**) *Halothiobacillus* HT1 biofilm reacted with CuS; (**Right**) *Halothiobacillus* HT1 biofilm.

XRF is considered to be a powerful tool for quantitative mapping of trace element distributions [16]. It can visualize the metal ion distribution in tissues or cells. The colony biofilms were thicker in the center and thinner at the edges. The thickness of the biofilm grown on CuS was about 150 μm, while the control was about 100 μm. Figure 2 showed the *Halothiobacillus* HT1 biofilms section, the scanning area of XRF and the distribution characters of the elements in the scanning area. The elements were not evenly distributed in the biofilms. There was a Cu accumulation layer in the middle of the *Halothiobacillus* HT1 biofilm in the presence of CuS, suggesting Cu penetrated about 100–150 μm from the membrane interface.

**Figure 3:**
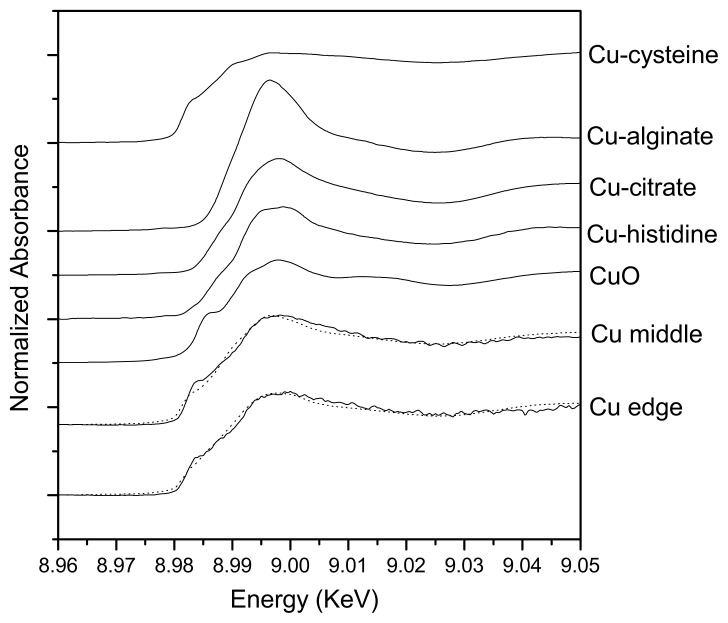
Comparison of copper K-edge XANES spectra of reference compounds and *Halothiobacillus* HT1 biofilm after being reacted with CuS. Black solid lines: data. Dashed lines: fits with data sets of reference compounds.

Compared with bulk XAFS, micro-XAFS could analyze the speciation of elements in small ozone. The Cu K-edge micro-XANES spectra were conducted to determine the speciation of Cu in the center and at the edge of the biofilm formed on CuS. Linear combination arithmetic was used to determine the probable Cu speciation in the sample as analyzed by the LSFitXAFS program. There were some differences in the XANES spectra of the center and the edge, as shown in Figure 3. As shown in Table 1, species resembling Cu-cysteine was the major species both in the center (67.8%) and at the edge (48.4%). In the center of the biofilm reacted with CuS, Cu-alginate- and Cu-citrate-like species accounted for 21.4% and 10.8%, respectively. While at the edge, Cu-histidine- (22.3%) and CuS (19.3%)-like species accounted for minor proportions, with Cu-alginate- and Cu-citrate-like species in smaller proportions (2.9% and 7.1%, respectively).

### 2.3. Sulfur Speciation in the Biofilms

**Figure 4:**
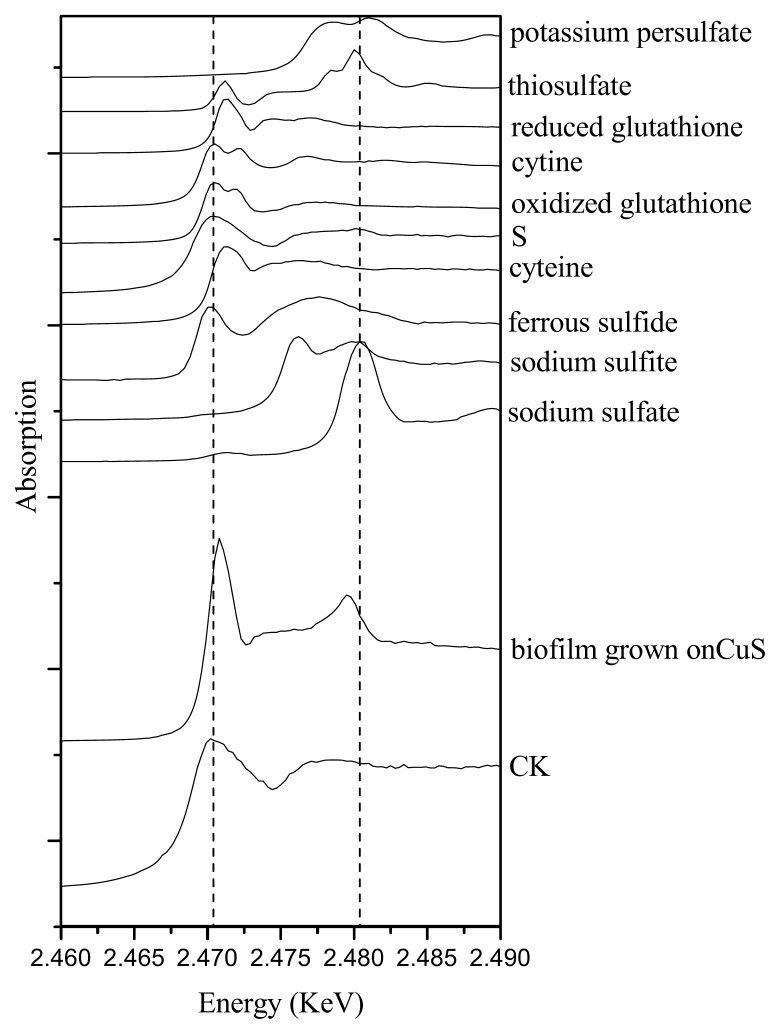
Comparison of sulfur K-edge XANES in *Halothiobacillus* HT1 biofilm (CK), *Halothiobacillus* HT1 biofilm reacted with CuS and reference compounds.

As an essential macronutrient for microorganisms, plants and animals, sulfur (S) exists in a variety of organic and inorganic species, with oxidation states ranging from −2 to +6. The spectra of reference compounds and the samples are shown in Figure 4. Different reference compounds presented different structures of S. For example, cysteine, reduced glutathione presented R–SH, while cystine, oxidized glutathione presented R–S–S–R. There were multiple oxidations of sulfur in biofilm formed on CuS. As shown in Table 2, species resembling cysteine (R–SH) were the major sulfur species in the biofilm (77.3%), with species resembling sulfite (SO_3_^2−^) and sulfonate (R–SO_2_–O–X) in minor proportions. While in the absence of CuS (the control sample), no oxidized sulfur was detected.

## 3. Discussion

### 3.1. The Relationship of Cu Distribution and Live/Dead Cells in Biofilms

Using CLSM, two active zones were found in the biofilm of HT1: the air-biofilm interface and the membrane-biofilm interface. Biofilm formation seemed to give rise to distinct chemical and redox gradients. The different distributions of the cells indicated that oxygen played an important role in the distribution of live cells [17]. At the air-biofilm interface, oxygen was enough for the growth of aerobes. As a result, *Halothiobacillus* HT1 cells in this area were active, leading to the larger quantities of live cells than in the middle. While at the membrane-biofilm interface, where the oxygen density was relatively low, more live cells were detected than in the middle. The reason might be attributed to the fact that this area played an important role in the transport of nutrition. Therefore, the cells were activated at the medium-biofilm interface. Biofilm growth of pathogenic bacteria might influence the rate of carbon and inorganic nutrient cycling [18]. The XRF results showed nonuniform distributions of the elements (Figure 4). Our results supported the previous reports that there were diverse niches in biofilms, and biofilms were physiologically distinct from bacteria in planktonic form [19–21]. The presence of gradients of dissolved gases, nutrients and solute concentrations might be responsible for the distribution of live and dead cells of *Halothiobacillus* HT1.

In addition, a better understanding of the distribution and mass transfer process within the biofilm is important for understanding their influences on biogeochemical transformation of metals. Metal might deposit as metallic oxide, phosphate, sulfate or carbonate at certain area, due to the heterogeneous distribution of pH, Eh and O_2_. As a micro-analytical technique for the quantitative mapping of elemental distributions, XRF is compatible with fully hydrated biological samples [22,23]. In this study, XRF is used to detect the distribution of Cu in biofilm of *Halothiobacillus* HT1 formed on CuS. Our results indicated that CuS can be oxidized by *Halothiobacillus* HT1, as oxidized sulfur (sulfite and sulfonate) was detected using sulfur K-edge XANES analysis (Table 2). As a result, ionic copper was obtained and penetrated into the *Halothiobacillus* HT1 biofilm. As shown in Figure 2, copper accumulated in the layer near the membrane-biofilm interface. This result indicated that the CuS and *Halothiobacillus* HT1 biofilm reacted actively. This might be another reason for the larger quantity of live cells in membrane-biofilm interface than in the middle. The heterogeneous distribution of Cu found in this study was similar with the result of Templeton conducted on the distribution of Pb at biofilm-metal oxide interfaces [8]. Another study also showed that Zn equally distributed in the 12 μm surface layer of the 35 μm *E. coli* biofilm [24]. All these results confirmed the heterogeneous distribution of metal in microbe biofilms. Synchrotron-based XRF and micro-XANES spectra were successfully applied to investigate the spatial distribution of elements within HT1 biofilms and the speciation of Cu. Our results indicated that there was spatial heterogeneity vertically (Figure 2) and horizontally (Figure 3 and Table 1) within the biofilms

### 3.2. Effects of *Halothiobacillus* HT1 Biofilms on CuS Transformation

Some specific properties of biofilms can be related to the heterogeneity of their structure and composition. In this study, biofilms formed on CuS were about 150 μm thick, while the control was only about 100 μm, indicating that HT1 might react with the CuS, since CuS provided substrate for it. To further analyze how microbes influence the fate of metal sulfide, speciation of Cu and S was studied. Using sulfur K-edge XANES, sulfur transformation in the biofilms was found. As shown in Figure 4, sulfur was oxidized to cysteine, sulfite and sulfonate in the biofilms formed on CuS. This might be attributed to the reaction of CuS and *Halothiobacillus* HT1. Sulfur oxidizing bacteria are considered to be capable of oxidizing mineral sulfide to sulfate. In this study, oxidized sulfur detected in the biofilm indicated that CuS was oxidized biologically in the presence of *Halothiobacillus* HT1 biofilms. However, S was found to exist as diverse molecular species (cysteine, sulfite and sulfonate), which might be the intermediate products of sulfide oxidization, but not the end product (sulfate). The presence of cysteine, sulfite and sulfonate in the biofilm indicated heterogeneity in the redox state and implied that intermediate sulfur compounds were available for metabolism by *Halothiobacillus* HT1.

Intimate organic-mineral associations are often the result of bacterial activity. Attached bacteria and adsorbed organic matter may interact with sorption processes on metal sulfide surface by changing the characteristics of the electrical double layer at the solid-solution interface, blocking surface sites or providing a variety of new sites for metal binding. Extracellular polymeric substances (EPS) were considered to enclose biofilms and contain many active groups. Micro-XANES is an element-specific spectroscopic tool to identify the local environment of a target atom within a sample. Therefore, we also studied the speciation of copper in the biofilm formed on CuS by using micro-XANES. There were several active groups, such as hydroxyl (−OH), sulfhydryl (−SH) and amino (−NH_2_), in the biofilms. The Cu-cysteine analog was the major Cu species. In correspondence, sulfur K-edge XANES results also confirmed that cysteine was the main sulfur species.

In order to survive heavy metals stress, microbes usually apply different mechanisms, such as biosorption, precipitation and complexation with sulfides, phosphates or carbonates. Active efflux or detoxification of metal ions by different transformations was found to be involved in many metal resistance systems [25]. For some bacteria, it was believed that inorganic polyphosphates and transport of metal phosphate complexes were involved in heavy metal tolerance [26–28]. Cu homeostasis proteins were also found in previous study [29]. In this study, cysteine analogs likely played an important role in the penetration of copper into the biofilm. The high percentage of Cu-cysteine-like species in the biofilm confirmed that the Cu bond to cysteine groups could be defenses against toxic copper. Complexation with –SH groups might contribute to the detoxification of Cu since –SH-containing groups were considered to be bioactive molecules, which can maintain Cu homeostasis through cation binding. These results were consistent with some previous reports concerning other bacteria, which showed that cellular proteins rich in thiol groups participated in the copper homeostasis [30].

At the biofilm-mineral interface, many critical processes occur. Our study attempted to study the interaction of CuS and *Halothiobacillus* HT1 biofilm at the micro scale. More work about the interfacial properties of the biofilm-mineral interface are needed in the future. For example, scanning transmission X-ray microscopy (STXM), which uses near-edge X-ray absorption spectroscopy (NEXAFS) as its contrast mechanism, can be further used to fully hydrate biological materials at a smaller scale (nanoscale).

## 4. Experimental Section

### 4.1. Biofilms Cultivation

*Halothiobacillus* HT1 was retrieved from −70 °C stock cultures by incubating on the LB agar plate at 28 °C for 48 h. Then, a single colony was suspended into 1 mL of sterile 0.9% NaCl. Zero-point-one grams of CuS (pretreated with 0.25 M EDTA, pH 8.0 for antioxidation and acetone for sulfide remover) and 1 mL sterile water were added in an EP tube to prepare the suspension. Then, 300 μL of this suspension was added on LB solid medium. Nuclepore^®^ polyester membranes (13 mm diameter, 0.1 μm pore, 6 μm thick, Whatman) were sterilized by immersion in 70% ethanol for 2 min individually, air dried and transferred with sterile forceps to the LB solid media surface. Three membranes were spaced in an equilateral triangular pattern and 3 μL of the *Halothiobacillus* HT1 suspension were transferred to the center of each membrane and then inoculated at 28 °C for 72 h. Control treatments without adding CuS were also prepared. Each treatment was prepared in four groups for staining, XRF analysis, sulfur XANES and Cu micro-XANES analysis, respectively.

### 4.2. Live/Dead Cells Distribution Using CLSM Image

Biofilms were stained with the Live/Dead BacLight Kit (Molecular Probes) to determine the distribution of Live/Dead cells. Three microliters of Syto-9 and 3 μL propidium iodide were suspended in 1 mL 0.9% saline to prepare the staining agents. The biofilms were placed on the staining agent for 30 min in the dark. Live cells of *Halothiobacillus* HT1 stain green, and dead cells train orange-red.

Then, the stained biofilms were cryoembeded and cryosected as follows: biofilms were placed on the chuck. Cuts perpendicular to the plane of biofilms were made using a low profile microtome blade (Paracu™, McCormick™ Scientific). Fifty micrometer-thick cross sections from the middle of each biofilms were placed on glass coverslips and examined using microscopy to confirm the thickness and structure of biofilms sections and then stored at −20 °C for analysis. The samples were examined using a Leica TCS SP5 confocal laser scanning microscope (CLSM) to detect the distribution of live/dead cells of *Halothiobacillus* HT1.

### 4.3. Distribution of Cu in the Biofilm by Using Synchrotron-Based X-Ray Fluorescence

Biofilms were sectioned as described above and placed on 3M adhesive tapes (Scotch, 810, 3M Company, St. Paul, MN, USA). X-ray fluorescence (XRF) analysis of the biofilms was performed at the XRF microprobe station (beam line 4W1B) of Beijing Synchrotron Radiation Facility (BSRF), Institute of High Energy Physics of China. The electron energy in the storage ring was 2.2 GeV, with a current range from 78 to 120 mA. The size of the exciting X-ray beam was 10 μm × 20 μm. XRF spectra were collected by a PGY Si (Li) solid detector, positioned at 90° to the beam line. The scanning points of the samples were selected and observed by a microscope. Spectra data were processed by the AXIL program to integrate the area of the element excited peak. Relative contents of elements were calculated by calibrating the peak area with electron current and normalized with Compton scattering intensity. PyMca [31] and origin 8.0 was used to analyze the distribution of the elements in the biofilms.

### 4.4. Copper Speciation Analysis Using Cu K-Edge Micro-XANES

Two points located in the edge and center regions of the *Halothiobacillus* HT1 biofilm grown on CuS (the size of exciting X-ray beam was 40 μm × 70 μm) were selected for Cu micro-XANES analysis. A 50 μm thick cross section was obtained using cryosectioning methods described above and placed on 3 M adhesive tape and then stored at −20 °C until analysis. The Cu K-edge micro-XANES spectra were measured at beam line 15 U of Shanghai Synchrotron Radiation Facility (SSRF). It was operated at 3.5 GeV with a current of 210–150 mA in the fluorescence mode for the energy range from 8.95 to 9.06 keV. The fluorescence XANES spectra were collected for 5 s dwell times and analyzed as described by the previous description [32].

### 4.5. Sulfur Speciation Analysis Using Sulfur K-Edge XANES

Each of the biofilms were freeze dried, mixed and ground for XANES analysis. Sulfur speciation in the biofilms was analyzed by X-ray Absorption Spectroscopy at Beijing Synchrotron Radiation Facility (BSRF). The biofilms samples were laid on 3M tape for analysis. The storage ring was operated at the energy of 2.5 GeV with Si (111) double crystals. Spectra were recorded at 4B7A beamline (medium X-ray beamline, 2100–6000 eV) and scanned at step widths of 0.3 eV in the region between 2420 and 2520 eV. Reference compounds were ferrous sulfide, elemental sulfur, potassium persulfate, sodium thiosulfate, reduced glutathione, cystine, oxidized glutathione, cysteine, dimethyl sulfone, sodium diphenylamine sulfonate, sodium dodecyl sulfate (SDS), sodium sulfite and sodium sulfate. The X-ray energy was calibrated with reference to the spectrum of the highest resonance energy peak of Na_2_SO_4_ at 2,480.4 eV. The data were analyzed as described by the previous description [33].

## 5. Conclusions

Our results indicated that biofilm formation of *Halothiobacillus* HT1 gave rise to distinct chemical and redox gradients, leading to diverse niches in biofilms. There were two active zones in the biofilm of HT1: the air-biofilm interface and the membrane-biofilm interface. Copper was mainly distributed near the membrane-biofilm interface. *Halothiobacillus* HT1 biofilm oxidized CuS. Cysteine was synthesized for tolerance against Cu. The Cu-cysteine analog was involved in chelation within strain HT1 when it interacted with Cu to survive heavy metals stress.

## Figures and Tables

**Table 1:** The results of fitting the micro-XANES spectra with a linear combination of the measured data sets of representative model compounds. Copper oxidation states were assigned according to the similarity of the absorption edge energies of reference compounds. Rss: residual sum of squares.

Samples	Cu-cysteine (%)	Cu-alginate (%)	Cu-citrate (%)	Cu-histidine (%)	CuO (%)	Rss
Center	67.8	21.4	10.8	-	-	0.51
Edge	48.4	2.9	7.1	22.3	19.3	0.51

**Table 2:** Percentage of different S species in the biofilms as assessed by S K-edge XANES (atom% S).

Treatments	Sulfur	Cysteine	Sulfite	Sulfonate
CK	100	0	0	0
CuS	0	77.3	3.8	18.9
